# Unravelling new pathways of sterol metabolism: lessons learned from in-born errors and cancer

**DOI:** 10.1097/MCO.0000000000000442

**Published:** 2018-02-01

**Authors:** Yuqin Wang, William J. Griffiths

**Affiliations:** Swansea University Medical School, ILS1 Building, Singleton Park, Swansea, UK

**Keywords:** bile acid, cholesterol, oxysterol

## Abstract

**Purpose of review:**

To update researchers of recently discovered metabolites of cholesterol and of its precursors and to suggest relevant metabolic pathways.

**Recent findings:**

Patients suffering from inborn errors of sterol biosynthesis, transport and metabolism display unusual metabolic pathways, which may be major routes in the diseased state but minor in the healthy individual. Although quantitatively minor, these pathways may still be important in healthy individuals. Four inborn errors of metabolism, Smith-Lemli-Opitz syndrome, cerebrotendinous xanthomatosis and Niemann Pick disease types B (NPB) and C (NPC) result from mutations in different genes but can generate elevated levels of the same sterol metabolite, 7-oxocholesterol, in plasma. How this molecule is metabolized further is of great interest as its metabolites may have an important role in embryonic development. A second metabolite, abundant in NPC and NPB diseases, cholestane-3β,5α,6β-triol (3β,5α,6β-triol), has recently been shown to be metabolized to the corresponding bile acid, 3β,5α,6β-trihydroxycholanoic acid, providing a diagnostic marker in plasma. The origin of cholestane-3β,5α,6β-triol is likely to be 3β-hydroxycholestan-5,6-epoxide, which can alternatively be metabolized to the tumour suppressor dendrogenin A (DDA). In breast tumours, DDA levels are found to be decreased compared with normal tissues linking sterol metabolism to cancer.

**Summary:**

Unusual sterol metabolites and pathways may not only provide markers of disease, but also clues towards cause and treatment.

## INTRODUCTION

In vertebrates, cholesterol can be synthesized by all cells from acetyl-CoA. Following cyclization of squalene to lanosterol via squalene-2,3-epoxide the pathway divides into two main routes known as the Bloch and Kandustch–Russell pathways leading to desmosterol and 7-dehydrocholesterol (7-DHC), respectively, as the immediate precursors of cholesterol [1]. Alternatively, cholesterol can be taken up by cells as lipoproteins and have a dietary origin. Cholesterol is an essential molecule to maintain membrane structure and is the metabolic precursor of bile acids and steroid hormones. It has also been suggested to be a signalling molecule in its own right [2^▪▪^]. 7-DHC is the precursor of 1α,25-dihydroxyvitamin D_3_, the biologically active form of vitamin D. Although the major pathways of cholesterol metabolism were delineated in the 20th century [3^▪^], recent studies have revealed new metabolic pathways from cholesterol and 7-DHC, generating metabolites with unexpected biological activity. 

**Box 1:**
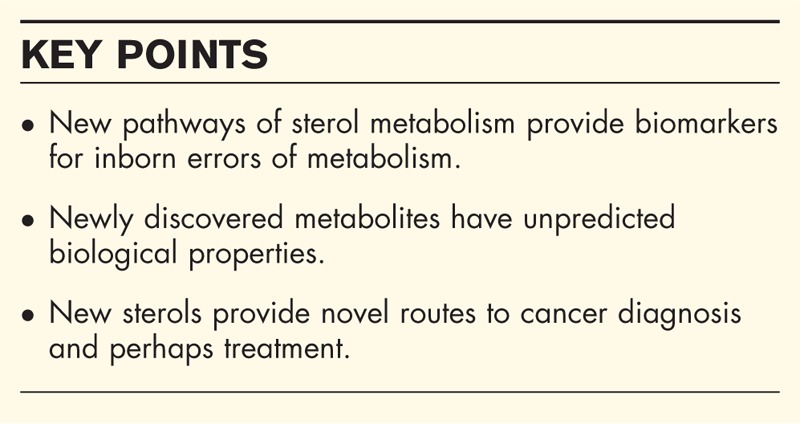
no caption available

## 7-OXOCHOLSTEROL

7-Oxocholesterol (7-OC), also known as 7-ketocholesterol, is a challenging sterol for biochemists to analyse as it may be formed by reaction of cholesterol with oxygen in air [4,5], but can also be formed endogenously via reaction of cholesterol with reactive oxygen species [6] or from 7-DHC enzymatically [7]. Many analytical scientists have been wary of reports of high levels of 7-OC in tissue and plasma, however, there is now convincing evidence that 7-OC is abundant in some disease states.

**FIGURE 1:**
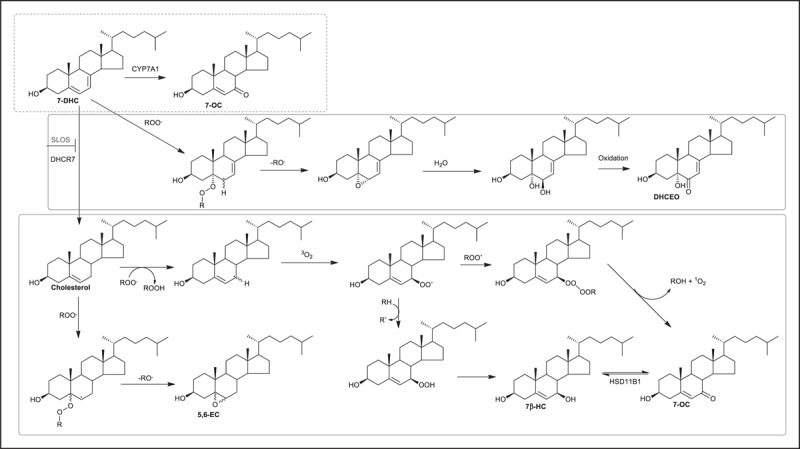
Enzymatic or nonenzymatic formation of 7-oxocholesterol, 3β,5α-dihydroxycholest-7-en-6-one and 3β-hydroxycholestan-5,6-epoxide. The pathways prevalent in SLOS are depicted in the red (upper) and blue (central) boxes, in CTX in the red (upper) box and in the lysosomal storage diseases NPB, NPC and LAL deficiency in the green (lower) box. The defective enzymatic step in SLOS is indicated by a horizontal T. ROO^.^, peroxy radical; RO^.^, alkoxy radical.

In agreement with earlier studies by Björkhem *et al.*[8], Pajares *et al.*[9] have reported elevated of 7-OC in plasma of patients suffering from cerebrotendinous xanthomatosis (CTX). They used a liquid chromatography (LC)–tandem mass spectrometry (MS/MS) method exploiting derivatization to *N,N-*dimethylglycine (DMG) esters and electrospray ionization (ESI). Levels of 7-OC in some CTX patients prior to treatment were as high as 1000 ng/ml (*n* = 11, mean 830 ng/ml, range 137– 529 ng/ml), compared with control values of about 10 ng/ml (adults *n* = 75, median 9.8 ng/ml, range 5.3–22.8 ng/ml, 5th to 95th percentile; children *n* = 32, median 13.8 ng/ml, range 8.3–34.5, 5th to 95th percentile). These values are for the free sterol as no hydrolysis step was carried out prior to analysis. CTX is an autosomal recessive disorder, where the enzyme cytochrome P450 (CYP) 27A1 is defective. People with CTX often develop neurological problems in early adulthood, which are thought to be caused by an abnormal accumulation of sterols and an increasing number of xanthomas in brain. In young patients CTX often present with liver disease. CYP27A1 is required for bile acid biosynthesis via the conventional pathways, introducing first an alcohol group and then a carboxylic acid to the terminal carbon of the sterol side-chain. We have also found 7-OC to be elevated in CTX plasma and speculate that this is a result of upregulation of CYP7A1, as consequence of reduced negative-feedback by primary bile acids and use of 7-DHC as the enzyme substrate [7,8] (Fig. 1).

Pajares *et al.*[9] also found 7-OC to be elevated in Niemann Pick disease type C (NPC, *n* = 16, range 178–795 ng/ml, 95% CI), lysosomal acid lipase (LAL) deficiency (*n* = 3, mean 77.7 ng/ml, range 29.6–178 ng/ml) and Smith-Lemli-Opitz syndrome (SLOS, *n* = 3). More recently, Boenzi *et al.*[10^▪^] have also found 7-OC to be elevated in patients with Niemann Pick type (NPB) and NPC, LAL deficiency and SLOS using LC-ESI-MS/MS, with derivatization to dimethylaminobutyric acid (DMAB) esters, again in the absence of saponification. Control levels of 7-OC were found to be 3.8–39.8 ng/ml (2.5th to 97.5th percentile) with a median of 16.1 ng/ml (*n* = 135), in NPC the median was 86 ng/ml (*n* = 16, range 21.9–963 ng/ml), in NPB only two patients were analysed where 7-OC was 62.8–383 ng/ml, in two LAL deficiency patients 7-OC was 35.5–103 ng/ml and in SLOS patients, the median was 139 ng/ml (*n* = 4, range 76.4–337 ng/ml).

SLOS is a congenital disease resulting from a defect in 7-dehydrocholesterol reductase (DHCR7), the final enzyme in the Kandustch–Russell pathway of cholesterol biosynthesis, resulting in elevated levels of 7-DHC in plasma and tissues. Patients with SLOS present with a broad phenotype ranging from autistic behaviour in mildly affected individuals to abnormalities in multiple organs, dysmorphology and failure to thrive in more severe cases [11]. In SLOS, elevated 7-OC can be explained by enzymatic conversion from abundant 7-DHC by CYP7A1 (Fig. 1) [7,8,12]. NPC, NPB and LAL deficiency are all lysosomal storage diseases [13^▪▪^]. In NPC and NPB, and perhaps LAL deficiency also, cholesterol accumulates in lysosomes. NPC has a variable age of onset, with a range of nonspecific neurological and psychiatric clinical features, it results from a defect in either NPC1 or NPC2 proteins required for the transport of nonesterified cholesterol from lysosomes [13^▪▪^]. NPB, also known as acid sphingomyelinase deficiencies (ASMDs), caused by mutations in the *SMPD1* gene, is believed to result from affected cholesterol transfer by NPC2 protein and presents with enlarged liver and spleen or spleen alone in early childhood. LAL deficiency results from defective LAL, the enzyme which hydrolyses cholesterol esters and triglycerides. Whenever LAL deficiency occurs in infants, it usually leads to death before 6 months of age; however, enzyme replacement therapy is now available [14^▪^]. Using LC-MS/MS, DMG derivatization and atmospheric pressure chemical ionization (APCI), Romanello *et al.*[15^▪^] analysed control (*n* = 60, median 27.08 ng/ml, inter quartile range, IQR, 24.31–30.66 ng/ml), NPC1 (*n* = 17, median 137.95 ng/ml, IQR 78.16–192.22 ng/ml) and NPB (*n* = 8, median 120.22 ng/ml, IQR 78.69–165.2 ng/ml) plasma samples for 7-OC. In agreement with Boenzi *et al.*[10^▪^], they concluded that although plasma levels of 7-OC could diagnose NPC, it could not differentiate NPC from NPB. Others have similarly found 7-OC plasma levels to be a diagnostic for NPC [16,17]. In NPC formation of 7-OC is likely to be by *in vivo* free radical oxidation [18^▪▪^] (Fig. 1), this is probably true for NPB and LAL deficiency also. A concern for the analytical chemist whenever measuring 7-OC is that it can also be formed from cholesterol *ex vivo*[5], this may explain some of the variation in control values in the three studies highlighted above. A better diagnostic would be an enzymatically formed metabolite of 7-OC that could only be formed endogenously.

**FIGURE 2:**
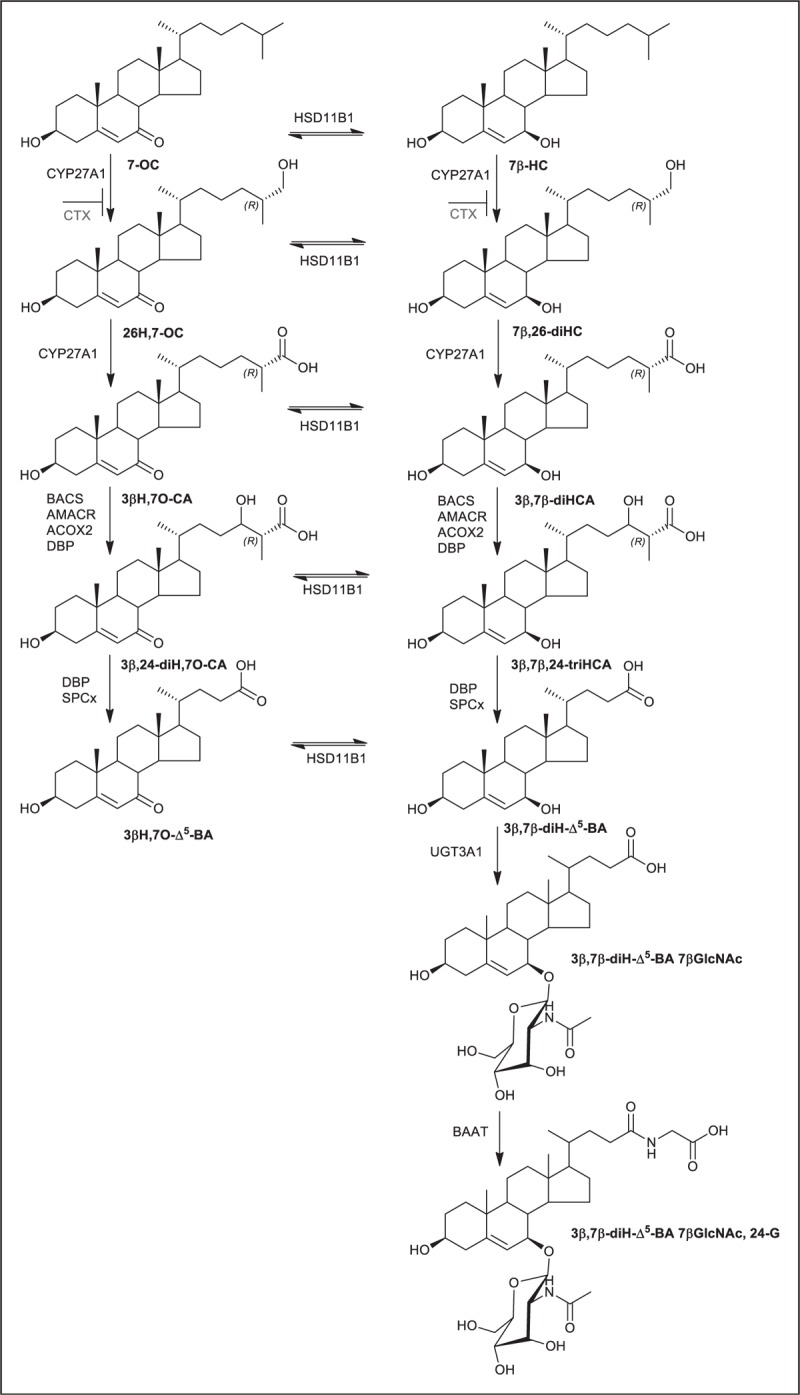
Metabolic transformation of 7-oxocholesterol to 3β,7β-diH-Δ^5^-BA 7β-GlcNAc and further conjugates in Smith-Lemli-Opitz syndrome and Niemann Pick disease type C. The defective enzymatic step in CTX is indicated by a horizontal T. ACOX2, acyl-coenzyme A oxidase 2; AMACR, alpha-methylacyl-CoA-racemase; BAAT, bile acid-CoA:amino acid *N*-acyltransferase; BACS, bile acyl CoA-synthetase; DBP, D bifunctional protein; SPCx, sterol carrier protein x; UGT3A1, UDP glycosyltransferase family 3 member A1.

Mazzacuva *et al.*[18^▪▪^] found 3β-hydroxy-7β-*N*-acetylglucosaminylchol-5-enoic acid (3β,7β-diH-Δ^5^-BA 7β-GlcNAc) to be elevated in NPC plasma and suggested its formation from 7-OC via 7β-hydroxycholesterol (7β-HC) (Fig. 2). We also suggested a pathway for the formation of this unusual bile acid involving conversion of 7-OC and its 7-oxo metabolites to 7β-hydroxy compounds by hydroxysteroid dehydrogenase (HSD) 11B1 and ultimate conjugation of the 7β-hydroxy group with *N*-acetylglucosamine (GlcNAc), a reaction known to be specific for the 7β stereochemistry (Fig. 2) [19]. Many years earlier we had found 3β-sulphated,7β-GlcNAc conjugated Δ^5^-bile acids modified with glycine or taurine in NPC urine and current studies in our laboratory indicate their formation in SLOS patients also [20]. 3β,7β-diH-Δ^5^-BA 7β-GlcNAc, and further conjugated forms, have potential as a biomarker for NPC and other disease states wherever 7-OC is elevated. However, Mazzacuva *et al.*[18^▪▪^] found a common mutation inactivating the GlcNAc transferase enzyme necessary for the formation of the GlcNAc conjugate. About 20% of Asian and Caucasian populations carry this mutation and fail to produce GlcNAc conjugates, hence, if these bile acids were to be used as a biomarker, many cases would be missed for NPC and also NPB, LAL deficiency and SLOS. However, the suggested biosynthetic pathway for 3β,7β-diH-Δ^5^-BA 7β-GlcNAc, particularly with respect to SLOS, does introduce some interesting metabolites [19]. One such metabolite is (25R)26-hydroxy-7-oxocholesterol (26H,7-OC), also called 27-hydroxy-7-ketocholesterol (Fig. 2). 26H,7-OC has been shown to bind to and activate the G protein-coupled receptor (GPCR) smoothened (SMO), which transmits signal across the plasma membrane in the Hedgehog (Hh) signalling pathway. Significantly, SLOS phenocopies dysregulated Hh signalling, and defective Hh signalling has been implicated in dysmorphology associated with SLOS [21^▪^]. In addition to 26H,7O-C, 3β,5α-dihydroxycholest-7-en-6-one (DHCEO) is a product of metabolism of 7-DHC in SLOS, in this case via the intermediate 7-dehydrocholesterol-5α,6α-epoxide (Fig. 1) [4]. DHCEO is an inhibitor of Hh signalling and we suggest that dysregulated formation of Hh-signalling pathway modulatory sterols during development is the cause of some of the phenotypical features of SLOS [19].

## 3β-HYDROXYCHOLESTAN-5,6-EPOXIDE AND CHOLESTANE-3β,5α,6β-TRIOL

**FIGURE 3:**
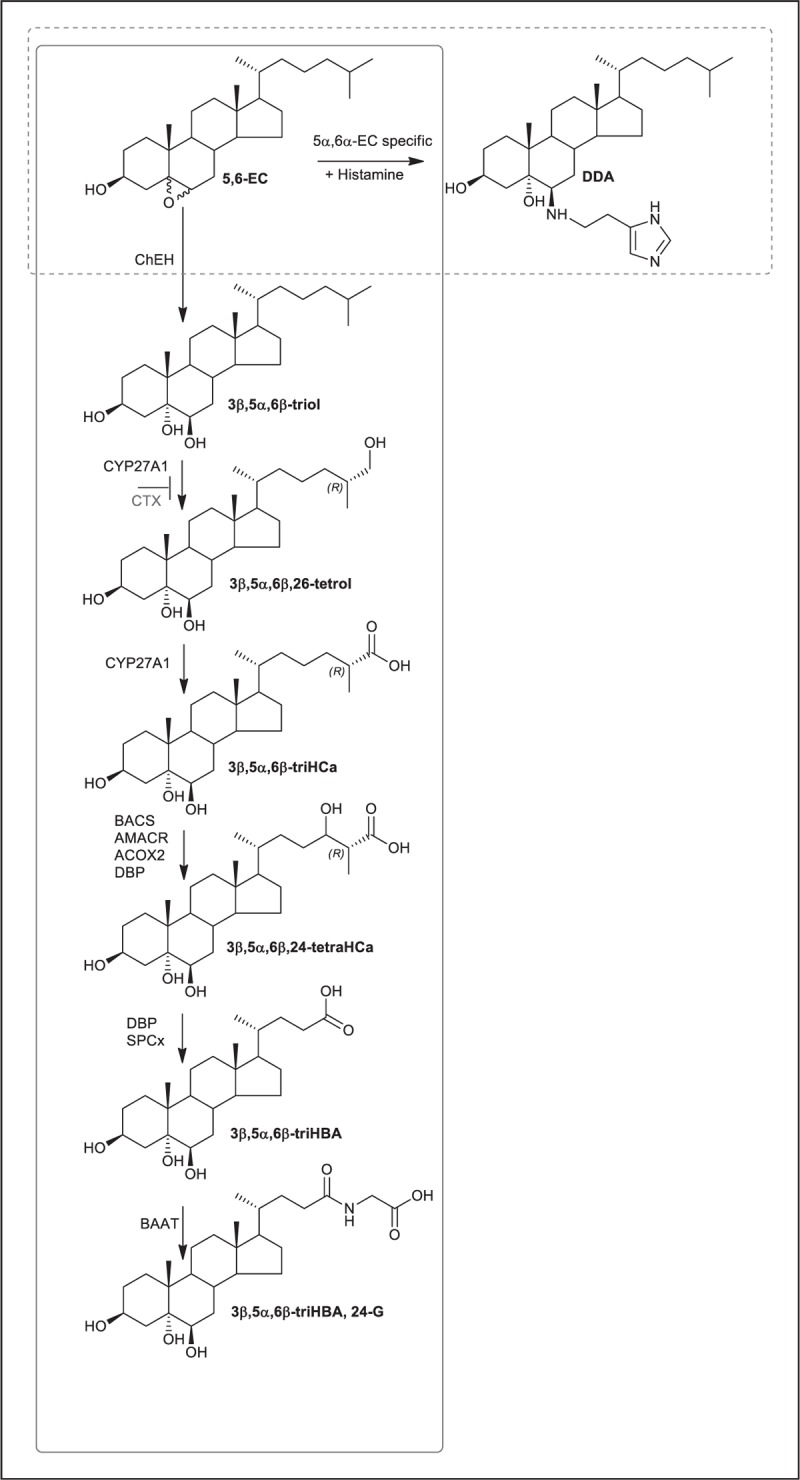
Metabolism of 3β-hydroxycholestan-5,6-epoxide to the unusual bile acid 3β,5α,6β-triHBA in Niemann Pick disease type B, Niemann Pick disease type C and lysosomal acid lipase deficiency (green box) or the histamine adduct dendrogenin A (purple box).

3β-Hydroxycholestan-5,6-epoxide (5,6-EC), also called 5,6-epoxycholesterol or cholesterol-5,6-epoxide, like 7-OC can be formed *ex vivo* from cholesterol oxidation in air and also *in vivo* through free radical reactions [4] (Fig. 1). To-date, no enzyme with cholesterol-5,6-epoxidase activity has been reported. However, the two isomers 5α,6α-EC and 5β,6β-EC can both be hydrolysed by cholesterol-5,6-epoxide hydrolase (ChEH) to cholestane-3β,5α,6β-triol (3β,5α,6β-triol) (Fig. 3) [22^▪▪^]. 5,6-EC can also be hydrolysed under acidic condition to 3β,5α,6β-triol during sample handling procedures. Like 7-OC, 3β,5α,6β-triol has been suggested as a plasma biomarker for NPC. Pajares *et al.*[9], Boenzi *et al.*[10^▪^] and Romanello *et al.*[15^▪^] have each found 3β,5α,6β-triol to be elevated in NPC plasma. Pajares *et al.* used the DMG derivative and LC-ESI-MS/MS. In addition to NPC plasma, 3β,5α,6β-triol was found to be elevated in plasma from patients with CTX and LAL deficiency. The median control plasma level of 3β,5α,6β-triol was 3.6 ng/ml (*n* = 107, range 0.5–8 ng/ml, 5th to 95th percentile), the mean CTX value was 43.7 ng/ml (*n* = 11, range 25.4–88.6 ng/ml), whereas NPC values ranged from 62 to 275 ng/ml (95% CI, *n* = 16) and LAL deficiency from 10.7 to 49.3 ng/ml (*n* = 3). SLOS patients (*n* = 3) were found to have normal levels of 3β,5α,6β-triol. Clearly, elevated plasma levels of 3β,5α,6β-triol are not unique to NPC. Romanello *et al.*[15^▪^] using a similar derivative and LC-APCI-MS/MS found plasma levels of NPC (median 48.44 ng/ml, IQR 24.86–60 ng/ml, *n* = 17) and also NPB (median 35.21 ng/ml, IQR 26.12–60.39 ng/ml, *n* = 8) elevated above control values (median 9.03 ng/ml, IQR 7.38–11.34 ng/ml, *n* = 60). This data shows that 3β,5α,6β-triol is elevated in both NPC and NPB. Boenzi *et al.* using DMAB derivatization and LC-ESI-MS/MS found control plasma levels of 3β,5α,6β-triol to have a median of 4.1 ng/ml in a range of 1.1–21.9 ng/ml (*n* = 135, 2.5th to 97.5th percentile). In NPC, the 3β,5α,6β-triol median was 55.3 ng/ml in a range 16–608 ng/ml (*n* = 16), in two NPB patients the range was 52–271 ng/ml, in two patients with LAL deficiency, the range was 22.8–45.1 ng/ml and in four SLOS patients the range was 1.7–7.4 ng/ml, similar to levels in control samples [10^▪^]. These three studies clearly indicate that elevated 3β,5α,6β-triol is not unique to NPC. Similar results have been found by others [23^▪▪^,^24^]. In a gas chromatography (GC)-MS study, Reunert *et al.*[23^▪▪^] analysed 1902 plasma samples from patients with a suspicion of NPC for 3β,5α,6β-triol. Diagnosis of patients with elevated 3β,5α,6β-triol was confirmed by genetic analysis. Twenty-four new mutations were identified in *NPC1*, one in *NPC2* and three in *SMPD1*, confirming the diagnostic potential of 3β,5α,6β-triol for the lysosomal storage diseases NPC and NPB.

As is the situation with 7-OC, *ex vivo* oxidation of cholesterol can lead to the formation of 5,6-EC, which may be subsequently hydrolysed to 3β,5α,6β-triol during sample handling procedures. Hence, elevated 3β,5α,6β-triol may be a consequence of sample handling and storage. Whenever 3β,5α,6β-triol is formed *in vivo*, it is likely to be metabolized further to a bile acid. In 2016, Mazzacuva *et al.*[18^▪▪^] and Jiang *et al.*[25^▪▪^] both reported the identification of elevated levels of the unusual bile acid 3β,5α,6β-trihydroxycholanoylglycine (3β,5α,6β-triHBA 24-G) in plasma of NPC patients. Mazzacuva *et al.* found the levels of this bile acid (median 118 ng/ml, *n* = 73) to be more than 10-fold higher than in controls (9.3 ng/ml, *n* = 84). Jiang *et al.* found levels of both the unconjugated and glycine-conjugated bile acid to be elevated in NPC plasma. They reported reference ranges for the glycine conjugate for controls of less than 5–5.34 ng/ml (*n* = 1013), NPC1 carriers of less than 5–12.5 ng/ml (*n* = 130) and NPC1 patients 5.45–294 ng/ml (*n* = 25). We have performed a similar study and find that the unconjugated bile acid 3β,5α,6β-trihydroxycholanoic acid (3β,5α,6β-triHBA) is also elevated in plasma from patients with NPB and LAL deficiency [26]. In our study, we identify 3β,5α,6β-trihydroxycholestanoic acid (3β,5α,6β-triHCa) and speculate that this acid is further metabolized to 3β,5α,6β-triHBA in the peroxisome (Fig. 3).

## DENDROGENIN A

Cholesterol-5,6-epoxide hydrolase (ChEH) will transform 5,6-EC to 3β,5α,6β-triol. Interestingly, the enzyme is made up of two subunits, 3β-hydroxysteroid-Δ^8,7^-isomerase (D8D7I) and DHCR7, and is identical to the microsomal protein complex antiestrogen-binding site (AEBS), which binds to tamoxifen, the anticancer drug, with high affinity. Inhibition of ChEH activity by tamoxifen binding induced cancer cell-differentiation through accumulation of 5,6-EC [22^▪▪^]. These findings lead Poirot and colleagues to search for a metabolite of 5,6-EC, other than those generated from 3β,5α,6β-triol, that may be display anticancer properties. They discovered dendrogenin A (DDA), a 6β-histamine adduct of 5α,6α-EC (Fig. 3) [22^▪▪^]. DDA was found to display anticancer properties *in vitro* and *in vivo*. DDA is found in mammalian tissues and at significantly lower concentrations in patients with breast tumours than normal matched tissue. Analysis of DDA is challenging. Its polar nature dictates analysis by LC-MS/MS rather than GC-MS, however, Noguer *et al.*[27], using LC-MS/MS, have experienced serious problems of carryover between chromatographic runs. This, however, can be solved by addition of heptafluorobutyric acid to the mobile phase. This breakthrough should now allow the discovery of downstream metabolites of DDA and perhaps another new metabolic pathway of cholesterol metabolism.

## CONCLUSION

In recent years the biological significance of nonenzymatically derived sterols has been realised. How they are metabolized is an area of great interest as newly discovered sterol metabolites may have unexpected biological activity.

## Acknowledgements

*Members of the European Network for Oxysterol Research (ENOR,*http://oxysterols.com/*) are thanked for informative discussions.*

### Financial support and sponsorship

This work was supported by the UK Biotechnology and Biological Sciences Research Council (BBSRC, grant numbers BB/I001735/1 and BB/N015932/1 to W.J.G., BB/L001942/1 to Y.W.) and the Welsh Government (to W.J.G. and Y.W.).

### Conflicts of interest

There are no conflicts of interest.

## REFERENCES AND RECOMMENDED READING

Papers of particular interest, published within the annual period of review, have been highlighted as:

▪ of special interest
▪▪ of outstanding interest

## References

[1] Griffiths WJ Abdel-Khalik J Hearn T Current trends in oxysterol research. Biochem Soc Trans 2016; 44:652–658. 27068984 10.1042/BST20150255 PMC4827129

[2▪▪] Luchetti G Sircar R Kong JH Cholesterol activates the G-protein coupled receptor smoothened to promote Hedgehog signaling. Elife 2016; 5: pii: e20304. 10.7554/eLife.20304 PMC5123864 27705744

[3▪] Vaz FM Ferdinandusse S Bile acid analysis in human disorders of bile acid biosynthesis. Mol Aspects Med 2017; 56:10–24. 28322867 10.1016/j.mam.2017.03.003

[4] Xu L Porter NA Free radical oxidation of cholesterol and its precursors: Implications in cholesterol biosynthesis disorders. Free Radic Res 2015; 49:835–849. 25381800 10.3109/10715762.2014.985219 PMC4461549

[5] Griffiths WJ Hearn T Crick PJ Charge-tagging liquid chromatography-mass spectrometry methodology targeting oxysterol diastereoisomers. Chem Phys Lipids 2017; 207 (Pt B):69–80. 28411018 10.1016/j.chemphyslip.2017.04.004 PMC5630687

[6] Nury T Zarrouk A Ragot K 7-Ketocholesterol is increased in the plasma of X-ALD patients and induces peroxisomal modifications in microglial cells: potential roles of 7-ketocholesterol in the pathophysiology of X-ALD. J Steroid Biochem Mol Biol 2017; 169:123–136. 27041118 10.1016/j.jsbmb.2016.03.037

[7] Guengerich FP Intersection of the roles of cytochrome P450 enzymes with xenobiotic and endogenous substrates: relevance to toxicity and drug interactions. Chem Res Toxicol 2017; 30:2–12. 27472660 10.1021/acs.chemrestox.6b00226 PMC5293730

[8] Björkhem I Diczfalusy U Lovgren-Sandblom A On the formation of 7-ketocholesterol from 7-dehydrocholesterol in patients with CTX and SLO. J Lipid Res 2014; 55:1165–1172. 24771866 10.1194/jlr.P048603 PMC4031947

[9] Pajares S Arias A Garcia-Villoria J Cholestane-3beta,5alpha,6beta-triol: high levels in Niemann-Pick type C, cerebrotendinous xanthomatosis, and lysosomal acid lipase deficiency. J Lipid Res 2015; 56:1926–1935. 26239048 10.1194/jlr.M060343 PMC4583089

[10▪] Boenzi S Deodato F Taurisano R Evaluation of plasma cholestane-3beta,5alpha,6beta-triol and 7-ketocholesterol in inherited disorders related to cholesterol metabolism. J Lipid Res 2016; 57:361–367. 26733147 10.1194/jlr.M061978 PMC4766985

[11] Boland MR Tatonetti NP Investigation of 7-dehydrocholesterol reductase pathway to elucidate off-target prenatal effects of pharmaceuticals: a systematic review. Pharmacogenomics J 2016; 16:411–429. 27401223 10.1038/tpj.2016.48 PMC5028238

[12] Griffiths WJ Abdel-Khalik J Crick PJ Sterols and oxysterols in plasma from Smith-Lemli-Opitz syndrome patients. J Steroid Biochem Mol Biol 2017; 169:77–87. 26976653 10.1016/j.jsbmb.2016.03.018 PMC5018427

[13▪▪] Vanier MT Gissen P Bauer P Diagnostic tests for Niemann-Pick disease type C (NP-C): a critical review. Mol Genet Metab 2016; 118:244–254. 27339554 10.1016/j.ymgme.2016.06.004

[14▪] Jones SA Rojas-Caro S Quinn AG Survival in infants treated with sebelipase Alfa for lysosomal acid lipase deficiency: an open-label, multicenter, dose-escalation study. Orphanet J Rare Dis 2017; 12:25. 28179030 10.1186/s13023-017-0587-3 PMC5299659

[15▪] Romanello M Zampieri S Bortolotti N Comprehensive evaluation of plasma 7-ketocholesterol and cholestan-3beta,5alpha,6beta-triol in an Italian cohort of patients affected by Niemann-Pick disease due to NPC1 and SMPD1 mutations. Clin Chim Acta 2016; 455:39–45. 26790753 10.1016/j.cca.2016.01.003

[16] Polo G Burlina A Furlan F High level of oxysterols in neonatal cholestasis: a pitfall in analysis of biochemical markers for Niemann-Pick type C disease. Clin Chem Lab Med 2016; 54:1221–1229. 26650075 10.1515/cclm-2015-0669

[17] Pataj Z Liebisch G Schmitz G Matysik S Quantification of oxysterols in human plasma and red blood cells by liquid chromatography high-resolution tandem mass spectrometry. J Chromatogr A 2016; 1439:82–88. 26607314 10.1016/j.chroma.2015.11.015

[18▪▪] Mazzacuva F Mills P Mills K Identification of novel bile acids as biomarkers for the early diagnosis of Niemann-Pick C disease. FEBS Lett 2016; 590:1651–1662. 27139891 10.1002/1873-3468.12196 PMC5089630

[19] Griffiths WJ Abdel-Khalik J Yutuc E Cholesterolomics: an update. Anal Biochem 2017; 524:56–67. 28087213 10.1016/j.ab.2017.01.009 PMC5378159

[20] Griffiths WJ Abdel-Khalik J Crick PT Bile acid biosynthesis avoiding cholesterol. In: XXIV International Bile Acid Meeting: Bile Acids in Health and Disease, 17–18 June 2016; Düsseldorf, Germany.

[21▪] Sever N Mann RK Xu L Endogenous B-ring oxysterols inhibit the Hedgehog component Smoothened in a manner distinct from cyclopamine or side-chain oxysterols. Proc Natl Acad Sci U S A 2016; 113:5904–5909. 10.1073/pnas.1604984113 PMC4889404 27162362

[22▪▪] Silvente-Poirot S de Medina P Record M Poirot M From tamoxifen to dendrogenin A: The discovery of a mammalian tumor suppressor and cholesterol metabolite. Biochimie 2016; 130:109–114. 27262406 10.1016/j.biochi.2016.05.016

[23▪▪] Reunert J Fobker M Kannenberg F Rapid diagnosis of 83 patients with Niemann Pick Type C disease and related cholesterol transport disorders by cholestantriol screening. EBioMedicine 2016; 4:170–175. 26981555 10.1016/j.ebiom.2015.12.018 PMC4776073

[24] Kannenberg F Nofer JR Schulte E Determination of serum cholestane-3beta,5alpha,6beta-triol by gas chromatography-mass spectrometry for identification of Niemann-Pick type C (NPC) disease. J Steroid Biochem Mol Biol 2017; 169:54–60. 26940355 10.1016/j.jsbmb.2016.02.030

[25▪▪] Jiang X Sidhu R Mydock-McGrane L Development of a bile acid-based newborn screen for Niemann-Pick disease type C. Sci Transl Med 2016; 8:337ra63. 10.1126/scitranslmed.aaf2326 PMC5316294 27147587

[26] Griffiths WJ Yutuc E Abdel-Khalik J Wang Y Metabolism of non-enzymatically derived oxysterols. In: Seventh ENOR SYMPOSIUM: Oxysterols and sterol derivatives in health and disease; 21–22 September 2017; Brussels.

[27] Noguer E Soules R Netter C Quantitative analysis of the tumor suppressor dendrogenin A using liquid chromatography tandem mass spectrometry. Chem Phys Lipids 2017; 207 (Pt B):81–86. 28684089 10.1016/j.chemphyslip.2017.06.010

